# Analyses of Antibacterial Activity and Cell Compatibility of Titanium Coated with a Zr–C–N Film

**DOI:** 10.1371/journal.pone.0056771

**Published:** 2013-02-19

**Authors:** Yin-Yu Chang, Heng-Li Huang, Chih-Ho Lai, Jui-Ting Hsu, Tzong-Ming Shieh, Aaron Yu-Jen Wu, Chao-Ling Chen

**Affiliations:** 1 Department of Mechanical and Computer-Aided Engineering, National Formosa University, Yunlin, Taiwan; 2 School of Dentistry, China Medical University, Taichung, Taiwan; 3 School of Medicine, China Medical University, Taichung, Taiwan; 4 Department of Dental Hygiene, China Medical University, Taichung, Taiwan; 5 Chang Gung Memorial Hospital, Chang Gung University College of Medicine, Kaohsiung, Taiwan; 6 School of Pharmacy, China Medical University, Taichung, Taiwan; Aligarh Muslim University, India

## Abstract

**Objective:**

The purpose of this study was to verify the antibacterial performance and cell proliferation activity of zirconium (Zr)–carbon (C)–nitride (N) coatings on commercially pure titanium (Ti) with different C contents.

**Materials and Methods:**

Reactive nitrogen gas (N_2_) with and without acetylene (C_2_H_2_) was activated by Zr plasma in a cathodic-arc evaporation system to deposit either a zirconium nitride (ZrN) or a Zr–C–N coating onto Ti plates. The bacterial activity of the coatings was evaluated against *Staphylococcus aureus* with the aid of SYTO9 nucleic acid staining and scanning electron microscopy (SEM). Cell compatibility, mRNA expression, and morphology related to human gingival fibroblasts (HGFs) on the coated samples were also determined by using the MTT assay, reverse transcriptase–polymerase chain reaction, and SEM.

**Results:**

The Zr–C–N coating with the highest C content (21.7 at%) exhibited the lowest bacterial preservation (*P*<0.001). Biological responses including proliferation, gene expression, and attachment of HGF cells to ZrN and Zr–C–N coatings were comparable to those of the uncoated Ti plate.

**Conclusions:**

High-C-content Zr–C–N coatings not only provide short-term antibacterial activity against *S. aureus* but are also biocompatible with HGF cells.

## Introduction

Among the metallic biomaterials, titanium (Ti) and its alloys have been used widely the field of biomedicine to manufacture orthopedic joints, bone screws and plates, and dental and orthodontic implants due to their excellent mechanical properties and biocompatibility [1]. However, Ti does not meet some of the clinical requirements of an implant biomaterial, such as exhibiting adequate antibacterial activity. The main causes of implant failure after the first year of occlusal loading are related to overloading [2], [3] and bacterial infection (e.g., peri-implantitis) [4]. Some researchers have found that biofilms induced by bacterial attachment have been responsible for approximately 65% of infections such as periodontal and peri-implant diseases. The role of peri-implant diseases induced by the bacteria around Ti implants has been recognized, and the antimicrobial properties of the Ti surface remain contentious [5]. One way of achieving better disinfection without affecting the biocompatibility of a Ti-based medical implant is to alter its surface properties.

Coating a medical implant surface with antibacterial agents exerts a beneficial effect by inhibiting bacterial attachment. Antibacterial agents for metallic medical implants can be classified into two groups: organic and inorganic [6]–[8]. Organic antibacterial agents contain antibiotics [9] and humanized antibodies [10], while inorganic antibacterial agents include silver (Ag), carbon (C), zinc (Zn), copper (Cu) [6]–[8], [11], and some oxide- and nitric-related coatings [(e.g., titanium dioxide, zinc oxide, tantalum nitride, titanium nitride (TiN), and zirconium nitride (ZrN)] [12]–[15]. One prerequisite of any antibacterial coating (organic or inorganic) is that it should not restrict tissue integration to the surface coating; rather, it would be beneficial if such coatings can in some way enhance that process.

The use of nitride coatings or films, such as ZrN, has been receiving attention due to their biocompatibility, hardness, erosion resistance, and antiwear properties [8]. However, the antibacterial properties of ZrN relative to those of Ti continue to be controversial [12], [16]. Like nitride hard coatings, amorphous C (a-C) films [also called diamond-like C (DLC) films] are also biocompatible, have high wear and corrosion resistances, chemical inertness, and a low coefficient of friction [8]. Some researchers have also reported obvious antibacterial properties of DLC films [17]. The application of medical devices coated with a-C has broadened in biomedical fields to include equipment such as orthopedic devices [18], [19].

While the inhibition of bacterial colonization by nitride coatings and a-C films has been confirmed, the effects of applying a zirconium (Zr)–C–nitride (N) coating to a Ti surface on the bacterial properties have not been reported, including for *Staphylococcus aureus*, which is reported to be associated with peri-implantitis [5], [20]. In the present study, Zr–C–N coatings with different C contents were synthesized using a cathodic-arc ion-plating process. The purpose of this study was to determine the effect of the C content of Zr–C–N coatings on the antibacterial performance and cytocompatibility of pure Ti in medical applications. The cell proliferation and gene expression of the Zr–C–N coatings was investigated by applying the MTT assay and reverse transcriptase–polymerase chain reaction (RT-PCR) analysis to human gingival fibroblast (HGF) cells cultured on the deposited samples.

## Materials and Methods

### Ethics Statement

HGF tissue was obtained from human gingival connective tissues that had been excised from patients during general teeth extractions. Permission to obtain the excess gingival tissue for experiments was obtained from each patient after he or she had read the written informed consent form (which was approved by the Ethics Committee of China Medical University Hospital, and collected name, age, gender, patient ID, and contact information; a legal representative agreement was also used to document the process). The gingival tissue could then legally be used for cell culturing. The entire procedure was approved by the Ethics Committee of China Medical University Hospital.

### Preparation for specimen coating

Three experimental conditions were employed: (1) pure Ti plates (surface roughness, Ra = 0.1 µm, biograde 2; Uniti Titanium, Moon Township, PA, USA), (2) ZrN-coated Ti plates, and (3) Zr–C–N-coated Ti plates. Each condition was tested by performing experiments on ten samples. Coatings were applied to the Ti plates using a cathodic-arc evaporation system. For ZrN and Zr–C–N coatings, circular Zr targets (100 mm in diameter) were arranged on the chamber wall to deposit the ZrN and nanocomposite Zr–C–N coatings. A DC arc current of 70 A was applied between the anode and the cathode using a high-current power supply (Miller XMT 304 CC/CV). The samples were placed onto a rotational substrate holder for the deposition of the ZrN and Zr–C–N coatings. Prior to deposition, a base pressure of less than 1×10^−3^ Pa was applied. Argon (Ar) and reactive gases [nitrogen (N_2_) and acetylene (C_2_H_2_)] were introduced via a duct around the target to enhance the reaction of the plasma during the deposition process. The temperature of the sample during the deposition was measured by a thermocouple located near the sample, and kept within the range of 100±20°C. A substrate bias voltage of −80 V was used. The ZrN coating was deposited at an N_2_ pressure of 1.5 Pa. For the deposition of various compositions of Zr–C–N film (i.e., Zr–5C–39N and Zr–22C–17N), ZrN was deposited as an interlayer (∼150 nm). At a total gas pressure of 1.5 Pa, a mixture of reactive N_2_ and C_2_H_2_ with different C_2_H_2_ flow rates of 10, 20, and 50 sccm was introduced into the chamber to form Zr–C–N coatings with different contents of C and N. The total thickness of the coatings was 0.5∼0.6 µm, as controlled by a fixed deposition time of 8 min.

The compositions of the deposited films were confirmed using X-ray photoelectron spectroscopy (XPS) (PHI1600 XPS system, Perkin-Elmer, Waltham, MA, USA) with nonmonochromatic magnesium Kα radiation. Ar ions were applied at 3 kV to sputter the surface oxide layer for 1 min to reveal the chemical composition of the deposited coatings. Survey spectra in the range of 0–1000 eV were recorded for each sample, followed by high-resolution spectra over different elemental peaks, from which the coating composition was calculated.

### Bacterial strain and cell culture

*S. aureus* (ATCC 29213), which was selected for testing the antibacterial activity, was recovered from frozen stocks on Brain Heart Infusion agar plates (Becton Dickinson, Franklin Lakes, NJ, USA), supplemented with 10% sheep blood and 1.5% agar, and incubated in a microaerophilic atmosphere at 37°C for 1–2 days. The *in vitro* antibacterial activity of each coated sample was evaluated.

The HGF cells were cultured in RPMI 1640 medium (HyClone, Logan, UT, USA) supplemented with 10% decomplemented fetal bovine serum (HyClone). Penicillin (100 U/ml) and streptomycin (100 mg/ml) (Invitrogen, Grand Island, NY, USA) were added to the culture medium. The HGF cells were then subjected to the MTT assay and RT-PCR analysis for each coated sample.

### Antibacterial test

The retention of bacteria on the coated samples was determined by a fluorescence-staining method employing SYTO9 nucleic acid stain (Molecular Probes, Eugene, OR, USA). First, 500 µl of an *S. aureus* suspension (2×10^7^ cfu/ml) was added to the sample surface. After incubation for 6 h at 37°C under a relative humidity of 96% and avoiding light exposure, the sample surfaces were rinsed three times with phosphate-buffered saline (PBS), and then any retained bacteria were fixed with 4% paraformaldehyde (Sigma-Aldrich, St. Louis, MO, USA) and stained with 10 µM STYO9 for 30 min at room temperature. The bacteria that had adhered to the coated samples were quantified by measuring the fluorescence detected at 488 nm by an enzyme-linked immunosorbent assay reader (Synergy HT, BioTek Instrument, Winooski, VT, USA). The results were determined from three independent experiments performed in duplicate, and quantified in units of relative fluorescence intensity.

### MTT assay and RT-PCR analysis

The proliferation of HGF cells was examined with an MTT assay (Sigma-Aldrich) after the cells were cultured on uncoated, ZrN-coated, or Zr–C–N-coated Ti surfaces. The substance used for the MTT assay was a 3-(4,5-dimethylthiazol-2-yl)-2,5-diphenyltetrazolium (MTT) salt, which turns into a purple formazan product in the presence of viable mitochondria in living cells. HGF cells (3 ml) were seeded at a density of 2×10^4^ cells/ml, and incubated at 37°C in 5% carbon dioxide (CO_2_) for 72 h, at which time proliferation was achieved. The MTT (5 mg/ml) was added to the cultured cells and incubated for a further 2 h. The purple formazan was eluted using 100 µl of isopropanol (Sigma-Aldrich). The absorbance of the purple formazan was quantified as the optical density (OD) measured at 570 nm by a SpectraMax spectrophotometer (Molecular Devices, Sunnyvale, CA, USA) with SoftMax Pro 5.2 241 software (Molecular Devices). The OD of formazan reflects the level of cell metabolic activity, with higher OD values indicating a larger number of living cells on the sample and hence better biocompatibility. Experiments were repeated independently in duplicate.

HGF cells (6×10^4^ cells) were cultured at 37°C in 5% CO_2_ for 72 h on the surfaces of pure Ti (control) plates, and on ZrN- and Zr–C–N-coated Ti plates. Total RNA was then isolated from the HGF cells using the TRIzol reagent (Invitrogen). The concentration of total RNA was determined by assessing its absorbance at 260 nm, and the quality of each sample was verified by inspection of the 18S and 28S ribosomal bands on an ethidium-bromide-stained agarose gel. Each sample (2 µg) was reverse transcribed into cDNA using an MMLV kit (Invitrogen). The oligonucleotide primers used corresponded to type I collagen (forward 5′-CTGGCAAAGAAGGCGGCAAA-3′ and reverse 5′-CTCACCACGATCACCACTCT-3′), type III collagen (forward 5′-GATATTGCACCCTATGACATTG-3′ and reverse 5′-GTTGAAGTTTATTTATTATAGCACC-3′), laminin (forward 5′-GAATCAGAATGGCTGGTAACATTTG-3′ and reverse 5′- CTAATGTGCCCAACTTCATTTCC-3′), fibronectin (forward 5′- GCCTGGTACAGAATATGTAGTG-3′ and reverse 5′- ATCCCAGCTGATCAGTAGGCTGGTG-3′), and glyceraldehyde-3-phosphate dehydrogenase (GAPDH; forward 5′-ACACCCACTCCTCCACCTTT-3′ and reverse 5′-TAGCCAAATTCGTTGTCATACC-3′). All oligonucleotide primers were synthesized by Invitrogen. The PCR reaction was performed as follows: 30 cycles of 95°C denaturation for 45 sec, 56°C (fibronectin, type I collagen, type III collagen, and GAPDH) or 52°C (laminin) annealing for 45 sec, and 72°C extension for 90 sec. The final elongation was conducted at 72°C for 60 sec. GAPDH mRNA served as an internal control for sample loading and mRNA integrity.

### Scanning electron microscopy

The retentive bacteria and attachment of HGF cells on the surfaces of uncoated and coated Ti samples were also examined using scanning electron microscopy (SEM). For specimens cultured with *S. aureus*, each sample was immersed in 3 ml of an aqueous solution of the bacteria (5×10^8^ cfu/ml) in lysogeny broth, and incubated therein for 4 h at 37°C. For specimens cultured with HGF cells, a 3-ml aqueous solution containing HGF cells was seeded onto the samples at a density of 2×10^4^ cells/ml, and incubated at 37°C in 5% CO_2_ for 24 h. The tested samples were rinsed three times with PBS and immediately fixed in 2.5% glutaraldehyde for 2 h. Prior to SEM, the tested samples were rinsed again with PBS, immersed in distilled deionized water for 10 min, and then dehydrated in an ethanol series (50%, 70%, 90%, 95%, and 100%; each for 10 min). The tested samples were fixed and subsequently dried by using critical-point drying with CO_2_, for which the critical point is at 31.1°C and 7.39 MPa, using a Samdri-PVT-3D apparatus. The chamber was precooled (10°C) to allow it to be readily filled with liquid CO_2_ from a gas cylinder. The chamber was then heated to just above the critical temperature, with critical pressure subsequently being achieved. Immediately after critical-point drying, samples were coated with platinum and then observed with the aid of a high-resolution, field-emission SEM device (7000F, Joel, Tokyo, Japan).

### Statistical analysis

The antibacterial activity and results of the MTT assay of uncoated pure Ti plates with ZrN- and Zr–C–N-coated samples were compared using Student's *t*-test. Differences were considered significant at *P*<0.001.

## Results

### Chemical composition data

The chemical compositions of the deposited ZrN and Zr–C–N (Zr–5C–39N and Zr–22C–17N) coatings were measured by XPS. The elemental composition of the ZrN coating was 56.7±1.2 at% Zr (mean±standard deviation) and 43.3±1.5 at% N. When C_2_H_2_ was introduced with N_2_ during the coating process, the Zr plasma generated by the arc evaporator reacted with the reactive gases (C_2_H_2_ and N_2_), allowing the C to substitute into the N lattice positions to form Zr–C–N. The compositions of the Zr–5C–39N and Zr–22C–17N coatings were 56.0±0.7 at% Zr, 5.3±0.5 at% C, and 38.7±1.3 at% N, and 61.7±1.0 at% Zr, 21.7±1.0 at% C, and 16.6±0.8 at% N, respectively. The bonding states of the deposited ZrN and Zr–C–N coatings were also characterized by XPS. This revealed that most of the C reacted with Zr to form a Zr–C–N solid solution during cathodic arc evaporation in this study. Amorphous C bonding was derived from the C1s core-level XPS spectra of the Zr–C–N coatings, which revealed a Zr–C–N coating with two coexisting metastable phases being synthesized, one of them being nanocrystalline Zr–C–N and the other being amorphous C.

### Antibacterial activity

**Figure 1:**
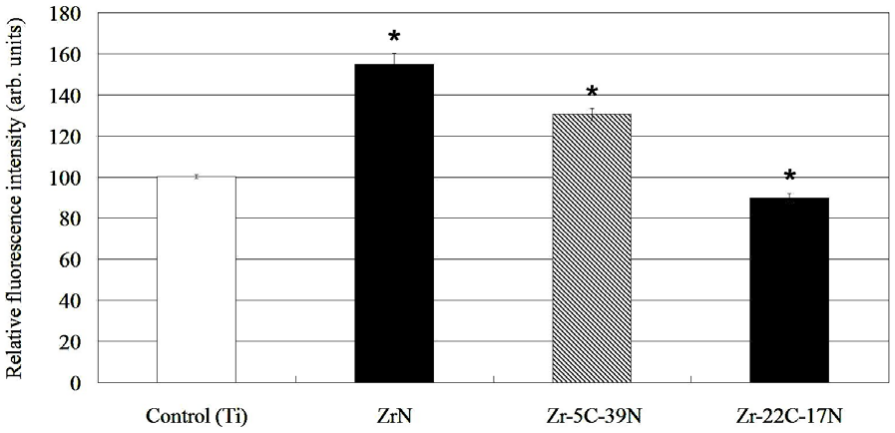
Antibacterial activity (in relative fluorescence units) of the uncoated Ti plates (control) and the ZrN-, Zr–5C–39N-, and Zr–22C–17N-coated Ti plates. Data are mean and standard deviation (SD) values of four independent experiments performed in duplicate. *Significantly different from control group (*P*<0.001).

**Figure 2:**
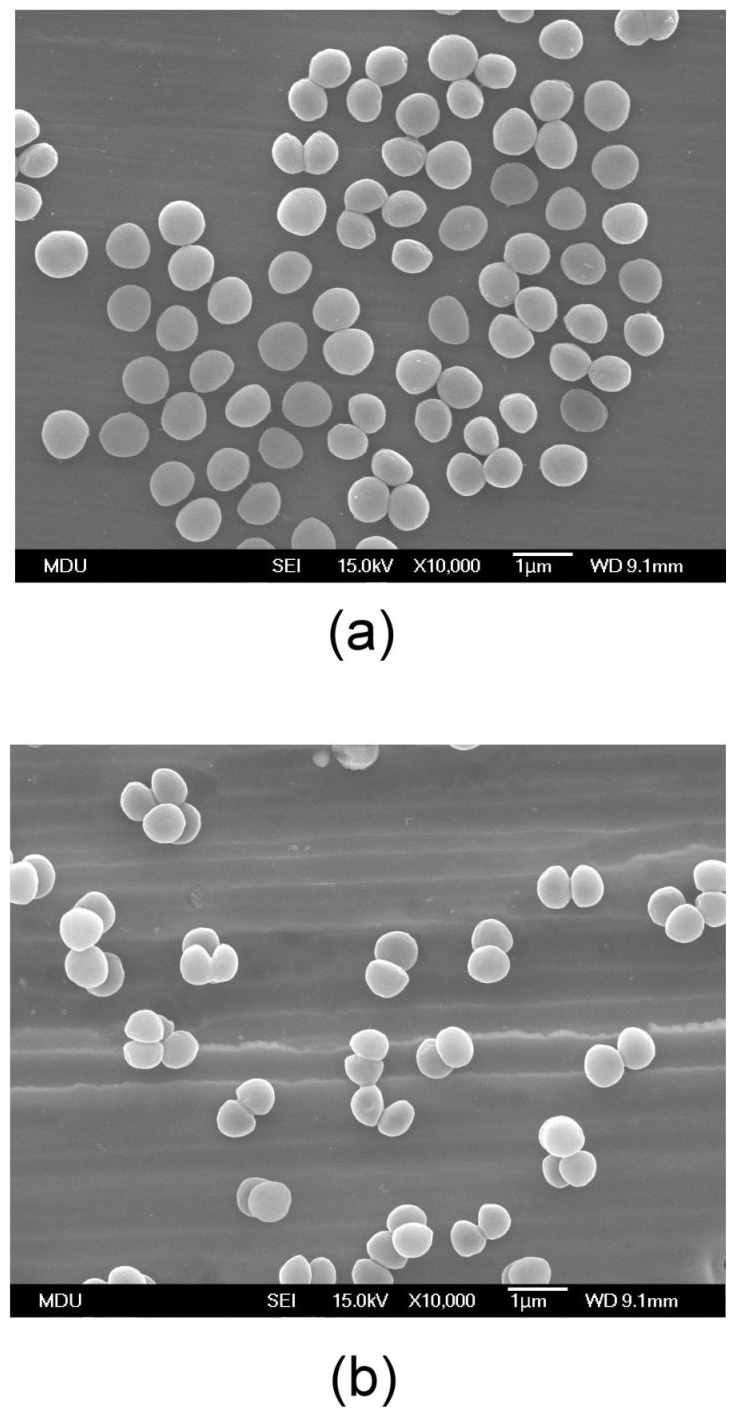
SEM micrographs of *S. aureus* incubated on (a) an uncoated Ti surface and (b) a Zr–22C–17N-coated Ti surface.

The relative fluorescence intensities of the *S. aureus* retained on control (Ti) plates, and ZrN- and Zr–C–N-coated Ti plates measured after 6 h by SYTO9 are shown in Fig. 1. The degree of bacterial preservation was higher on ZrN- and Zr–5C–39N-coated Ti plates than on the control sample (*P*<0.001), but it was significantly lower for the Zr–22C–17N-coated plates than for all of the other coatings (*P*<0.001). SEM images (Fig. 2) revealed that only a few *S. aureus* bacteria still adhered to and were scattered over the surface of Zr–22C–17N-coated plates, with considerably more bacteria observed on the uncoated Ti plates. These findings suggest that the Zr–22C–17N coating slightly but significantly enhances the *S. aureus* antibacterial activities of Ti-based materials.

### HFG cell proliferation and attachment

**Figure 3:**
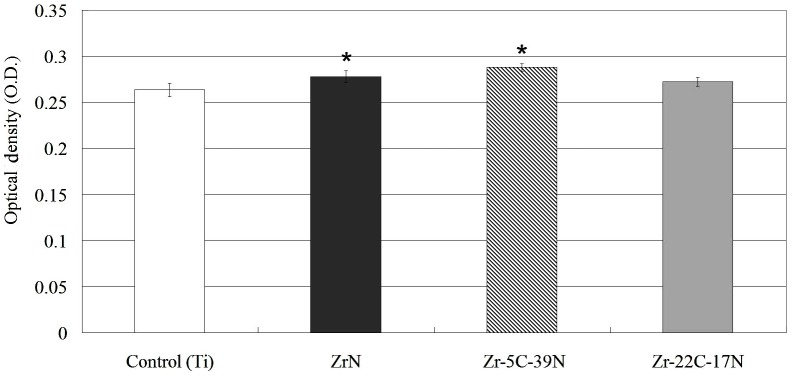
Cell proliferation test of HGF cells after 72 h of incubation with the uncoated Ti (control) plates and the ZrN-, Zr–5C–39N-, and Zr–22C–17N-coated Ti plates. Data are mean and SD values. *Significantly different from the control group (*P*<0.001).

The absorbance values of formazan (measured as OD via the MTT assay) by HGF cells for the control (Ti) sample and the ZrN- and Zr–C–N-coated specimens are shown in Fig. 3. The OD values were slightly but significantly higher for ZrN- and Zr–5C–39N-coated samples than for the control (Ti) sample (*P*<0.001). No significant difference was found between the data for the Ti and Zr–22C–17N-coated Ti plates.

**Figure 4:**
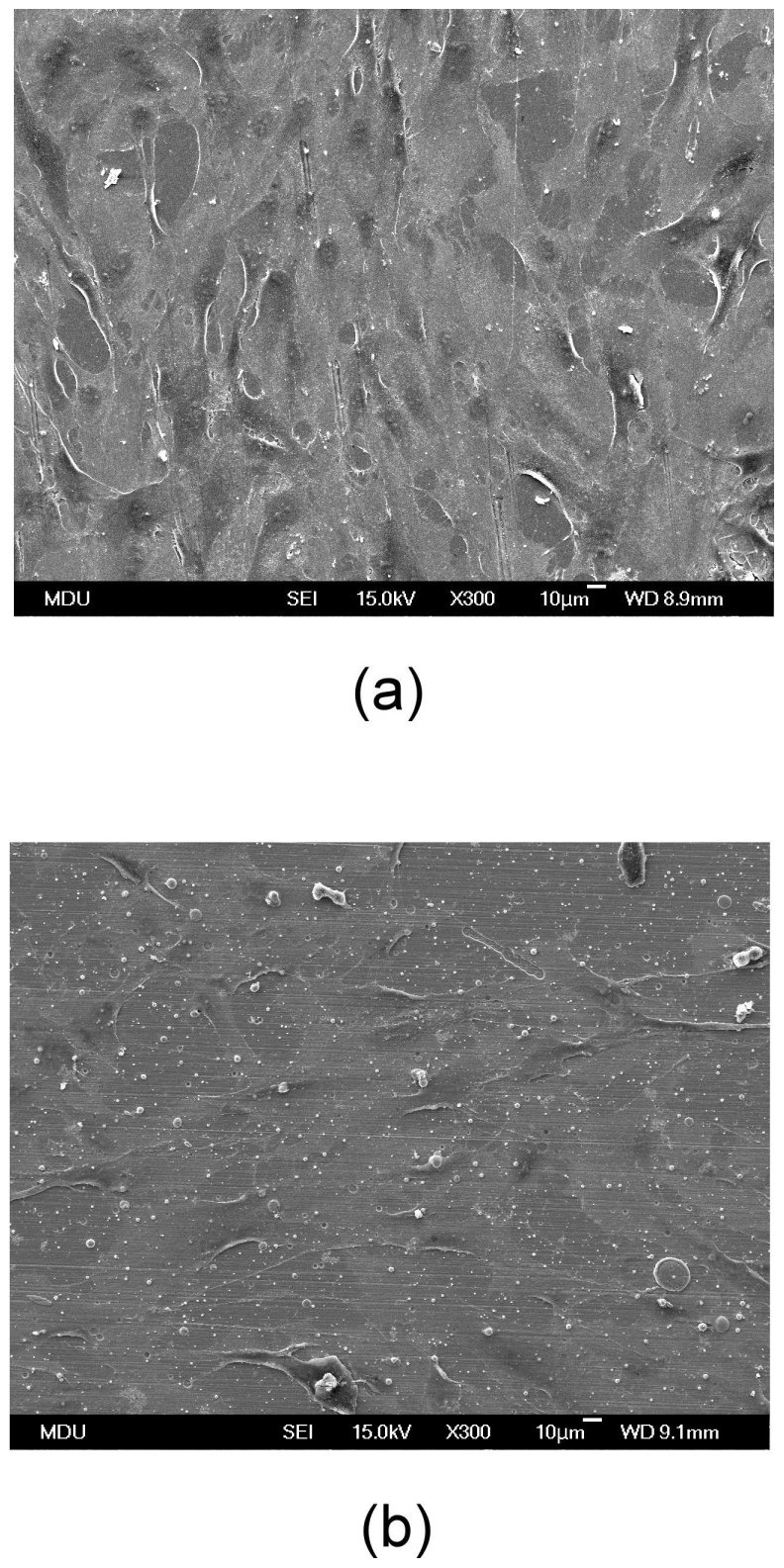
SEM micrographs of HGF cells cultured on (a) an uncoated Ti surface and (b) a Zr–22C–17N-coated surface.

The general shape and growth patterns of the HGF cells on the control sample and Zr–22C–17N-coated specimen are shown in Fig. 4. The HGF cells were well spread over and attached not only to the control samples but also to the Zr–22C–17N-coated specimens.

### HGF cell gene expression

**Figure 5:**
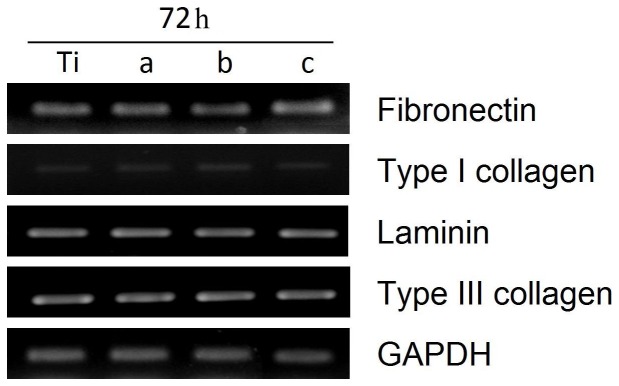
RT-PCR measurements of mRNA levels for fibronectin, type I collagen, laminin, type III collagen, and GAPDH. Columns a, b, and c represent ZrN, Zr–5C–39N, and Zr–22C–17N coatings, respectively.

After 72 h of incubation of HGF cells on each sample, the mRNA expressions of fibronectin, type I collagen, laminin, type III collagen, and GAPDH were analyzed by RT-PCR (Fig. 5). The level of mRNA for type I collagen in HGF cells was low on each sample. In addition, there appeared to be no significant differences between the levels of mRNA for fibronectin, laminin, type III collagen, and GAPDH in cells on the control and the ZrN- and Zr–C–N-coated specimens.

## Discussion

*S. aureus* is considered to be one of the more common causes of implant-associated infections [21] and peri-implantitis [5], [20]. Soft-tissue barriers such as the skin and mucous membranes can protect against a local tissue offensive by *S. aureus*; however, physical damage to these barriers by trauma or surgery can facilitate the entry of *S. aureus* into the underlying tissue and allow it to create its characteristic local abscess lesion. *S. aureus* can cause septicemia if it gets into the blood or lymphatic system [22]. Once *S. aureus* adheres to a metal surface it will form a biofilm, after which it can be difficult to treat clinically because biofilms protect the bacterial community from competing microorganisms [23]. Moreover, biofilms contribute to the spread of antibiotic resistance [24], [25]. One possible solution that may help to prevent the initial bacterial adhesion is to modify the surface chemistry of the metallic implant by using an antibacterial coating. The present study tested the putative antibacterial effects of two types of Zr–C–N coating and a ZrN coating against *S. aureus*.

The ZrN coating offered no advantage compared to Ti alone, since it did not prevent the growth of *S. aureus* on the surface of the Ti plate. This finding concurs with the results of Kertzman et al. [16], who found that after 72 h of bacterial culture, more bacteria had grown on the surface of ZrN-coated plates than on uncoated Ti plates. There are differing opinions regarding the antibacterial properties of nitride coatings. Some studies have indicated that certain types of nitride coating (e.g., TiN and titanium oxynitride) can minimize the adhesion of bacteria [12], [26], [27]. Different compositions of nitride films created by different physical-vapor-deposition coating techniques (e.g., cathodic-arc evaporation, sputtering deposition, and ion implantation) may influence the outcome of bacterial growth and adhesion, and so further investigations of the different types of films are warranted.

Using Ag and Cu as additional antibacterial agents in a nitride coating produces excellent antibacterial properties [16], [28], [29]. The present study revealed that C can also inhibit bacterial activity. One possible reason for the antibacterial performance of these C-based films is their chemical inertness inhibiting chemical interactions between the bacteria and the plate surface, and hence weakening the bacterial adhesion. Wang et al. [30] showed that the chemical bond between C and bacteria plays an important role in bacterial adhesion. The findings of some studies also suggest that C aggregates damage bacteria by physical damage to the outer membranes of the cells [31].

In the present study, the biological responses of HGF cells—including their proliferation and attachment to ZrN- and Zr–C–N-coated Ti plates—were comparable to those observed on uncoated Ti plates. This is consistent with the *in vivo* study of Groessner-Schreiber et al. [32] finding that Ti, TiN, and ZrN films favor the cellular adhesion of HGFs.

Types I and III collagens are major extracellular matrix components of fibroblasts, laminin and fibronectin are proteins involved in cell attachment [33], and the GAPDH gene actively regulates cell proliferation [34]. Our RT-PCR data revealed that the mRNA levels for these proteins and the GAPDH gene after 72 h of culture did not differ between the uncoated Ti plates and the ZrN- and Zr–C–N-coated samples.

Other studies have demonstrated that the use of ZrN coatings can maintain the biocompatibility of a biomaterial; moreover, the addition of certain antibacterial agents (e.g., C) to these films can inhibit bacterial activity relative to uncoated Ti plates [30], [31]. However, the present study involved the analysis of only one type of bacterium, and thus the obtained results only partially characterize the surface bacterial response for that specific bacterium. Since many bacterial species play a role in the etiology of peri-implantitis [35], future studies should include the use of different or mixed bacterial colonies [36], [37] of species related to peri-implantitis.

## Conclusions

ZrN- and Zr–C–N-coated Ti plates exhibited comparable HGF cell viability and proliferation to the uncoated Ti plates. The Zr–C–N coating with the highest C content (21.7 at %) had the lowest bacterial retention and exhibited a short-term antibacterial effect. In summary, the application of Zr–C–N coatings with a high C content not only slightly increased the antibacterial ability against *S. aureus* but also met the requirement of HGF cell biocompatibility.
